# Supplementary material for: Floquet control of interactions and edge states in a programmable quantum simulator
Source: Nat Commun. 2025 Oct 3;16:8815. doi: 10.1038/s41467-025-62897-2 (PMC12494747; doi:10.1038/s41467-025-62897-2)
Supplement: Supplementary file 1 — Supplementary Information [file 41467_2025_62897_MOESM1_ESM.pdf]

CONTENTS

List of Figures **1**

LIST OF FIGURES

1 **Supplementary Figure 1.** 2
2 **Supplementary Figure 2.** 3
3 **Supplementary Figure 3.** 4
4 **Supplementary Figure 4:** 5
5 **Supplementary Figure 5.** 6
6 **Supplementary Figure 6.** 7
7 **Supplementary Figure 7.** 8
8 **Supplementary Figure 8.** 9
9 **Supplementary Figure 9.** 10
10 **Supplementary Figure 10.** 11

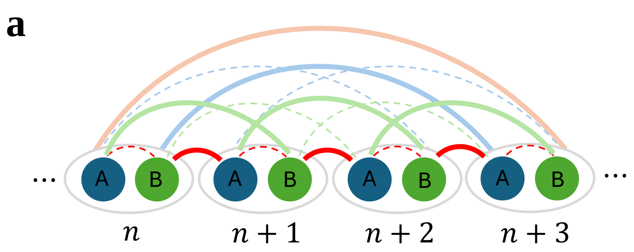

Supplementary Figure 1. **Sub-lattice structure of the long-range SSH spin model**. We label the odd (even) sites of a spin chain as sub-lattice A (B). The dimerization pattern of long-range couplings among different sublattices (**a**), and within the same sublattice (**b**) is shown for the case $\phi = \frac{3\pi}{4}$ which supports edge states. Lines represent the Dimerization parameter $\bar{D}$ (left) and D (right); see Eqs. (8-9). Weak bonds, suppressed by the Floquet fields and weighted by a dimerization parameter $j_0(\bar{\eta} z_0)$ are shown as dashed lines, while strong unsuppressed bonds are shown as continuous lines.

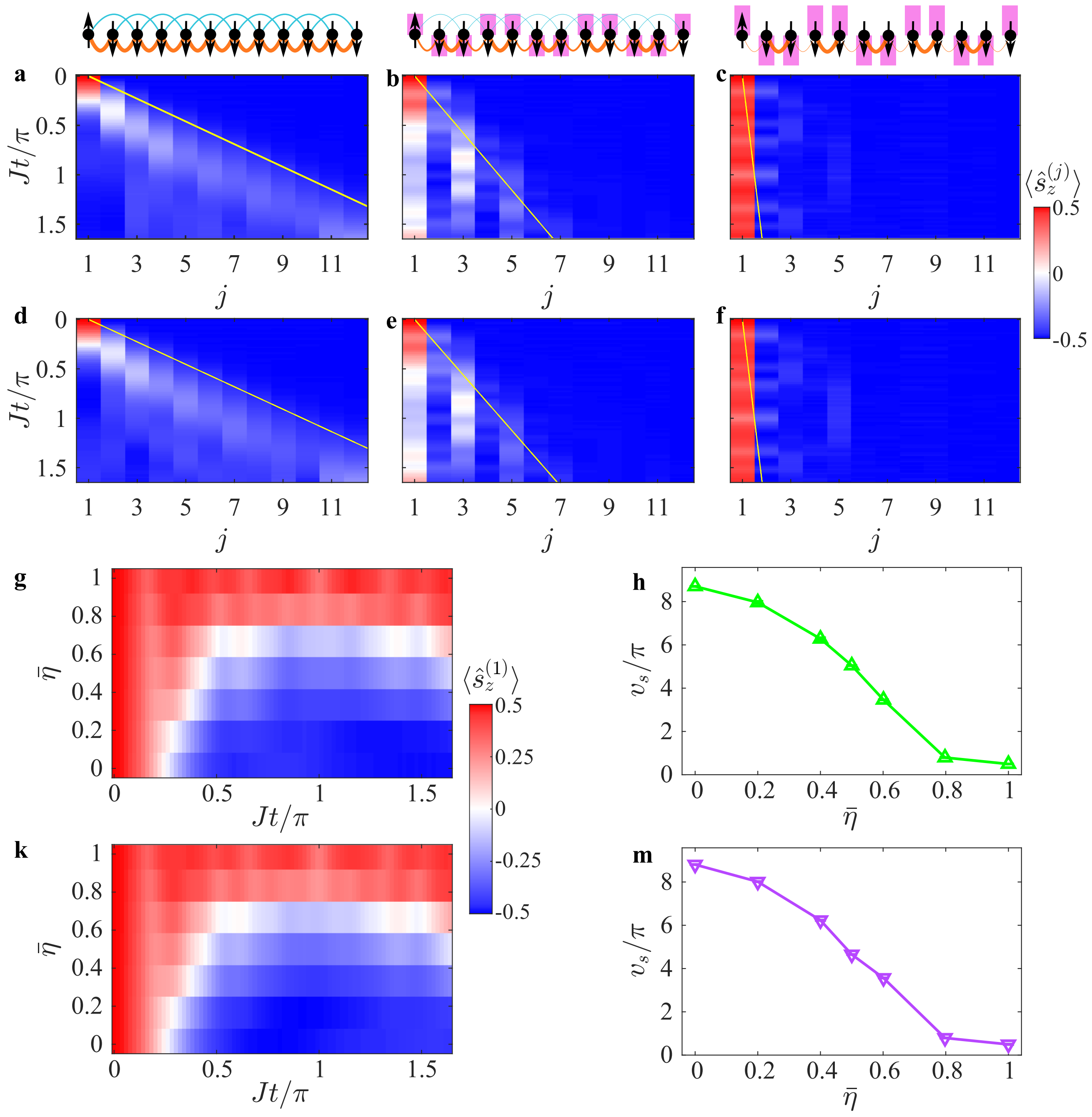

Supplementary Figure 2. **Numerical calculation of edge-excitation**. We numerically calculated the evolution of a single spin excitation at $j = 1$ for a $L = 12$, reproducing numerically the measurements presented in Fig. 1**d-h**. Plots **a,b,c,g,h** are computed using the time-dependent Hamiltonian in Eq. (2), while plots **d,e,f,k,m** use the modified SSH Hamiltonian in Eq. (1).

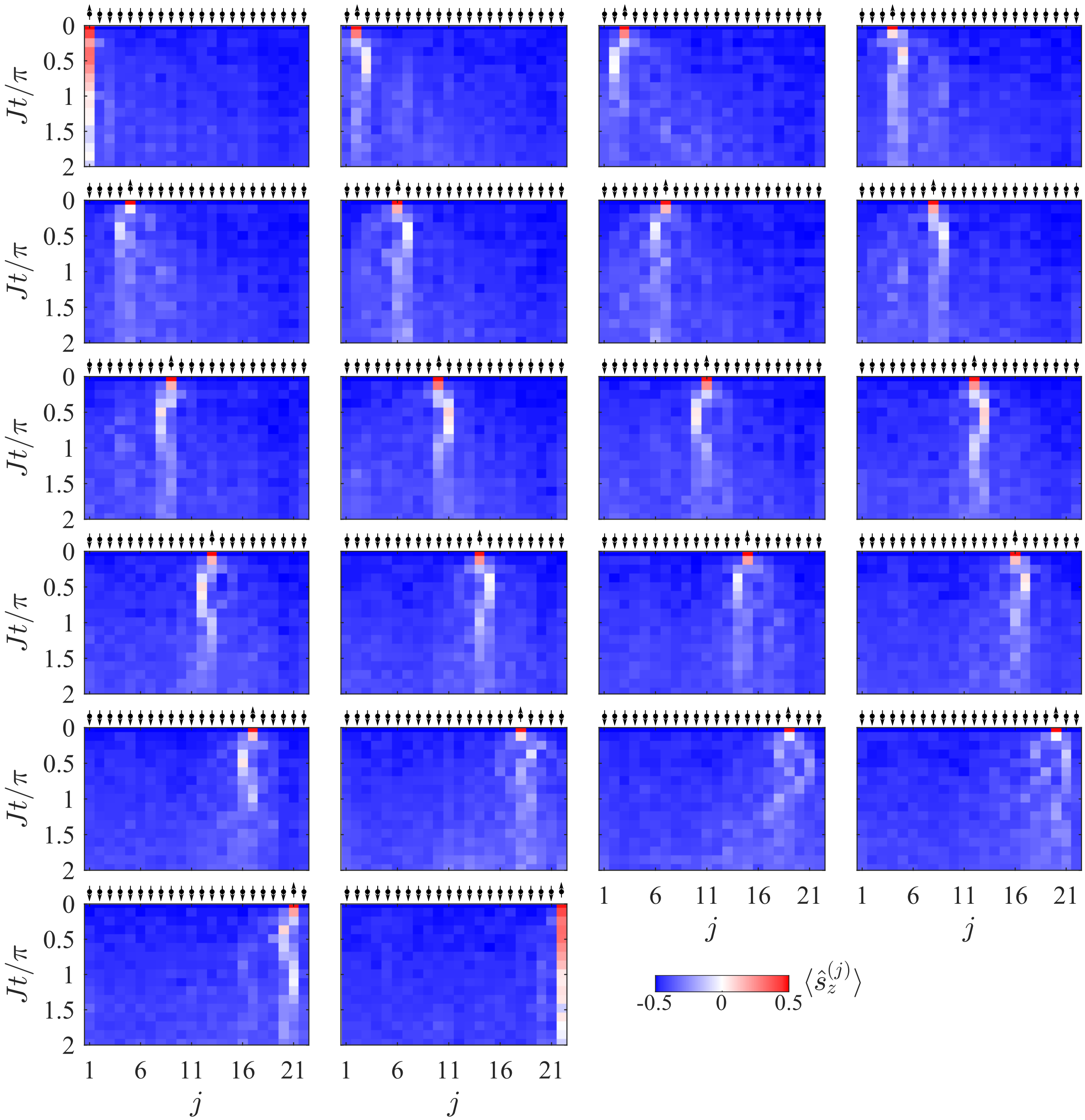

Supplementary Figure 3. **Single-Spin Excitation in an $L = 22$ Crystal**. Evolution of a single spin excitation over time for strong Floquet modulation with $\bar{\eta} = 0.8$. J represents the average nearest-neighbor spin bond coupling absent the Floquet drive. Each subplot, denoted as **a-v**, corresponds to one of the 22 experiments where the jth site is initially excited, with $1 \leq j \leq 22$.

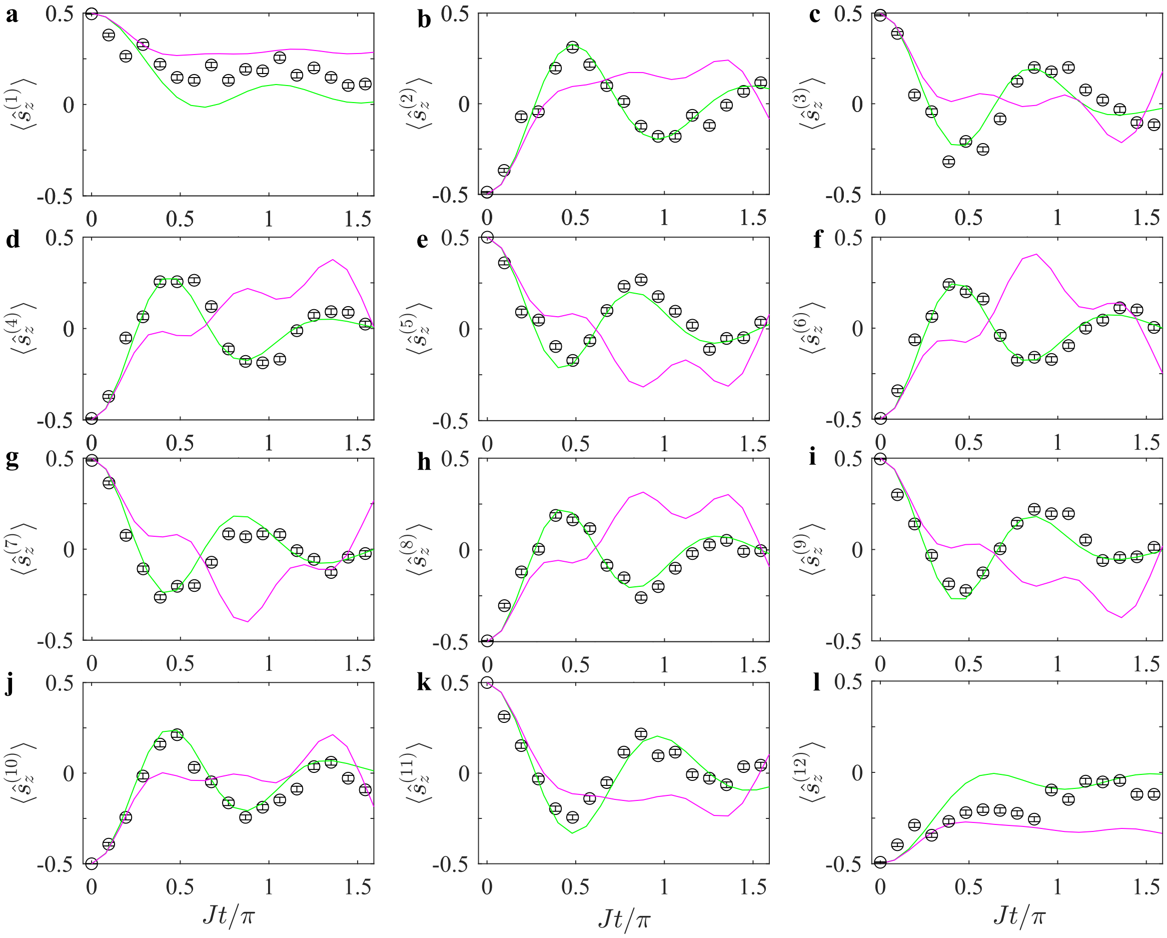

Supplementary Figure 4. **Dynamics of spins in Fig. 3b of the main text.** Dynamics for the $\bar{\eta} = 0.6$ configuration (Fig. 3**b**) shown for spins at sites $j = 1$ (**a**), $j = 2$ (**b**), $j = 3$ (**c**), $j = 4$ (**d**), $j = 5$ (**e**), $j = 6$ (**f**), $j = 7$ (**g**), $j = 8$ (**h**), $j = 9$ (**i**), $j = 10$ (**j**), $j = 11$ (**k**), $j = 12$ (**l**). Experimental data (black circles with error bars) agree with the numerically computed evolution under the full Hamiltonian (Eq. (1) in the main text, green curves), which is equivalent to a fermionic Hamiltonian including interaction terms (see text). For bulk spins ($2 \leq j \leq 11$), the data deviates from predictions of a long-range free-fermionic Hamiltonian (magenta curves).

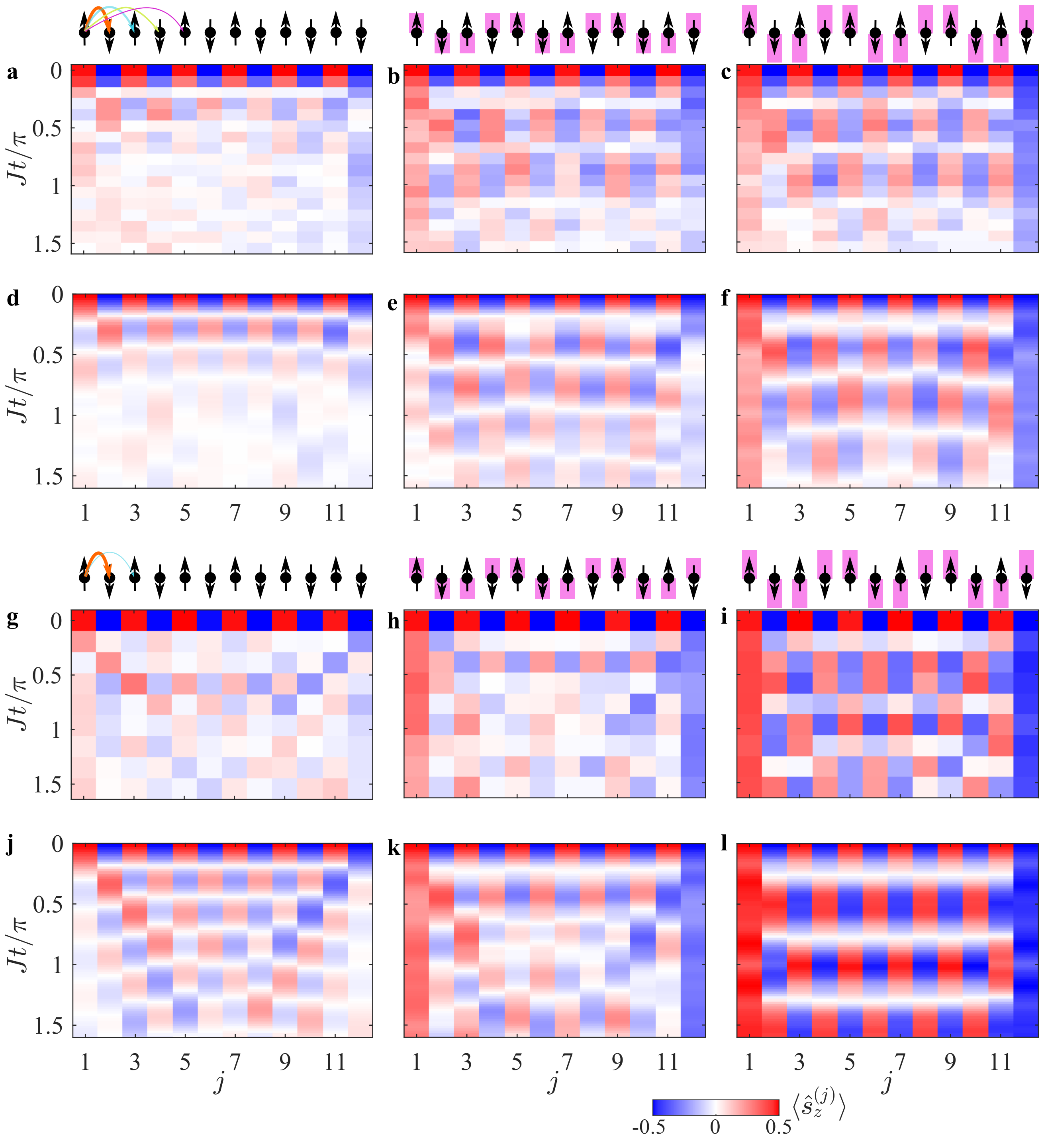

Supplementary Figure 5. **Evolution of staggered spin state**. We initialize the $L = 12$ crystal in the Néel spin state $|\uparrow\downarrow\uparrow\downarrow \ldots \uparrow\downarrow\rangle$ along the z axis and present the experimentally measured evolution for **a-c** long-range spin-spin interaction, **g-i** short range spin-spin interaction. The numerically calculated evolution (Eq. (2)) for the same configurations is depicted for the long range interaction in **d-f** and for short range interaction in **j-l**. **a,d,g,j** absent the Floquet drive ($\bar{\eta} = 0$), the spins thermalize quickly as excitations hop. In the presence of Floquet fields, $\bar{\eta} = 0.6$ in **b,e,h,k** and $\bar{\eta} = 1$ in **c,f,i,l**, the thermalization is suppressed and neighboring sites exchange exitations more efficiently while the edges remain isolated. For long range interaction, the suppression of thermalization is partial, and cannot be explained with a minimal model including only free-fermionic terms (see text).

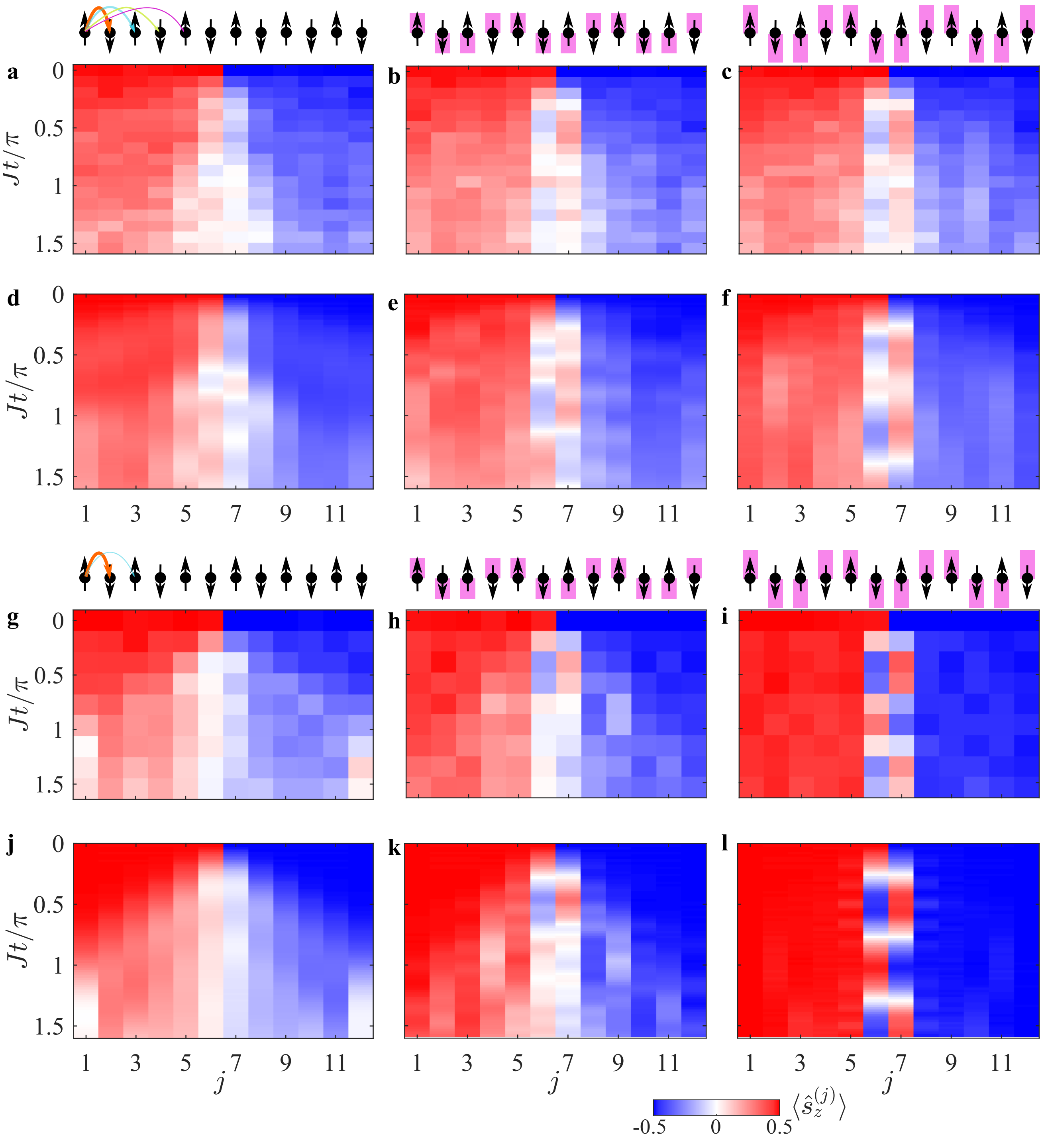

Supplementary Figure 6. **Evolution of domain wall**. We initialize the $L = 12$ crystal in the two-domain state $|\uparrow\uparrow\uparrow\uparrow\uparrow\uparrow\downarrow\downarrow\downarrow\downarrow\downarrow\downarrow\rangle$ along the z axis and present the experimentally measured the evolution for **a-c** long-range spin-spin interaction, **g-i** short range spin-spin interaction. The numerically calculated evolution (Eq. (2)) is depicted for the long range interaction in **d-f** and for short range interaction in **j-l**. **a,d,g,j** absent the Floquet drive ($\bar{\eta} = 0$), the spins thermalize quickly as excitations hop through the boundary. In the presence of Floquet fields, $\bar{\eta} = 0.6$ in **b,e,h,k** and $\bar{\eta} = 1$ in **c,f,i,l**, the thermalization is suppressed and spins at the boundary between the two domains exchange excitations more efficiently.

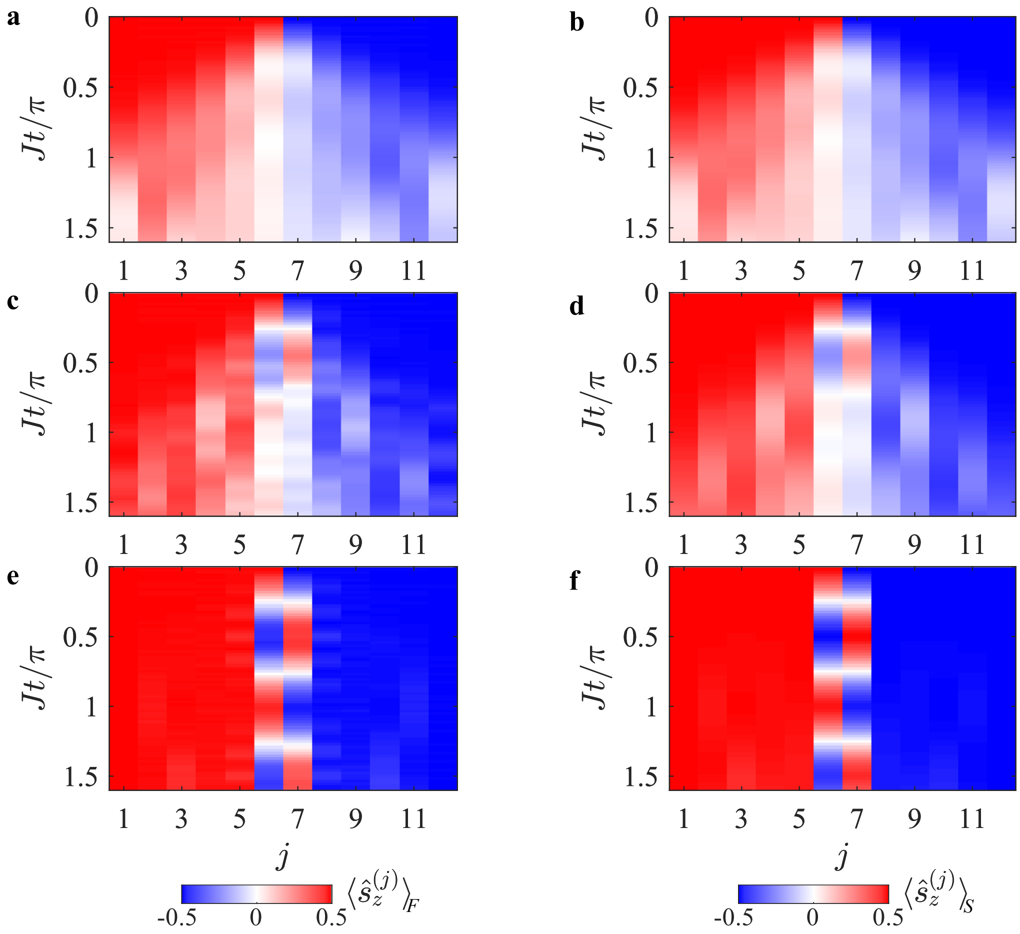

Supplementary Figure 7. **Comparison of SSH spin Hamiltonian and time-dependent Hamiltonian for the domain-wall configuration**. **(a,c,e)**: Numerical simulation of the short-range spin dynamics using the full time-dependent Hamiltonian (with time dependent Floquet fields), corresponding to subfigures **(j,k,l)** in Supplementary Figure 6. **(b,d,f)**: Numerical simulation using the static SSH Hamiltonian in Eq. (1) for the same parameters. Both models yield similar results, indicating that the contribution of fast-oscillating terms to the dynamics is minimal under our experimental conditions.

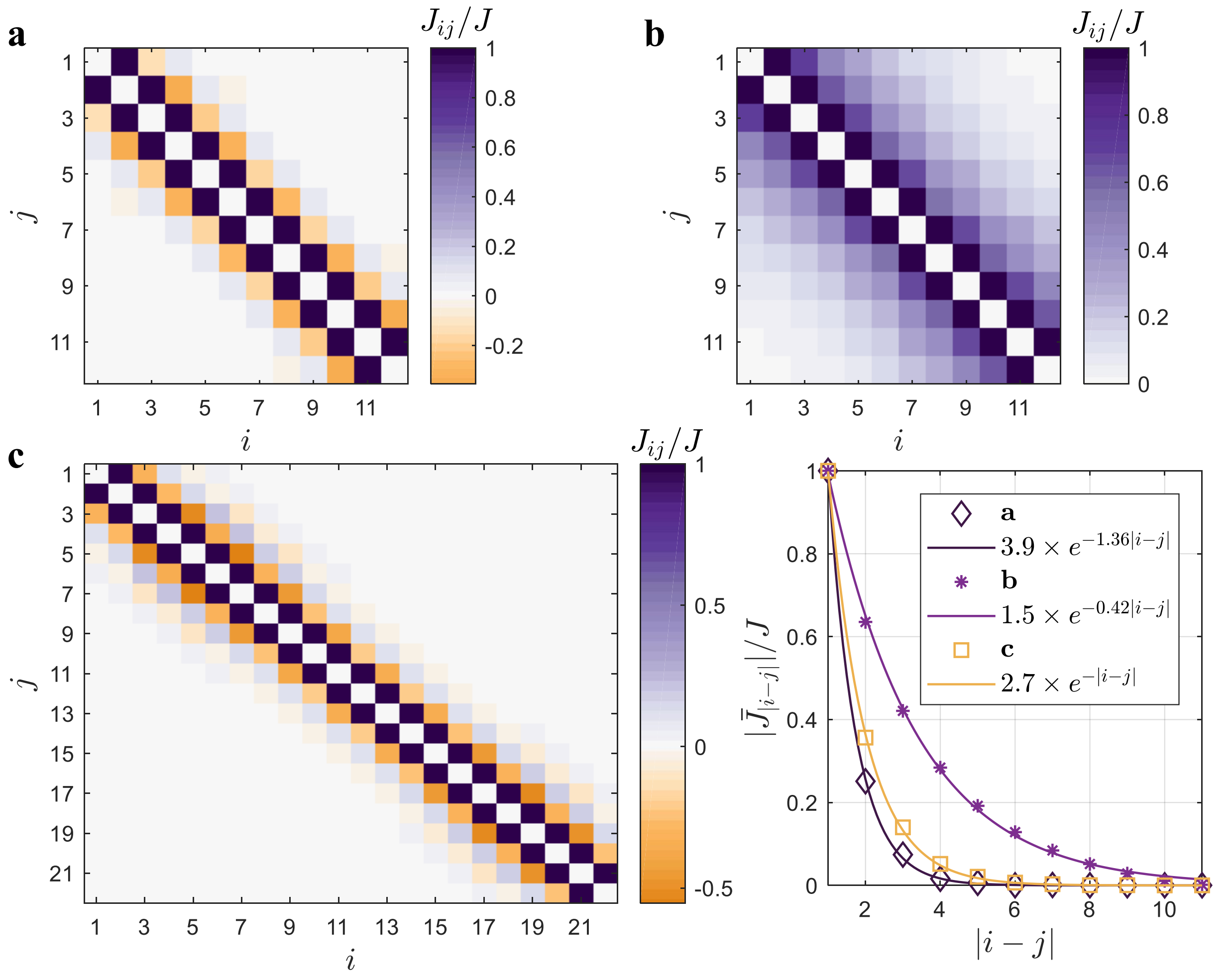

Supplementary Figure 8. **Calculated Spin-Spin Interaction Matrix**. **a-b** calculated interaction matrix for the $L = 12$ crystal. **a**, Short-range interaction. **b**, Long-range interaction. **c**, The interaction matrix for the $L = 22$ spin crystal. The staggered sign in **a,c** originates from the detuning near the zig-zag phonon modes (and are corrected via transformation, see Methods), while the positive sign in **b** results from detuning near the center of mass mode. These matrices are calculated using Eq. (4) and the parameters provided in the text. **d** Average bond strength between spins i and j at a distance $|i-j|$ for the three configurations, normalized by the average nearest neighbor bond strength J. Exponential fits provide approximations for the interaction range.

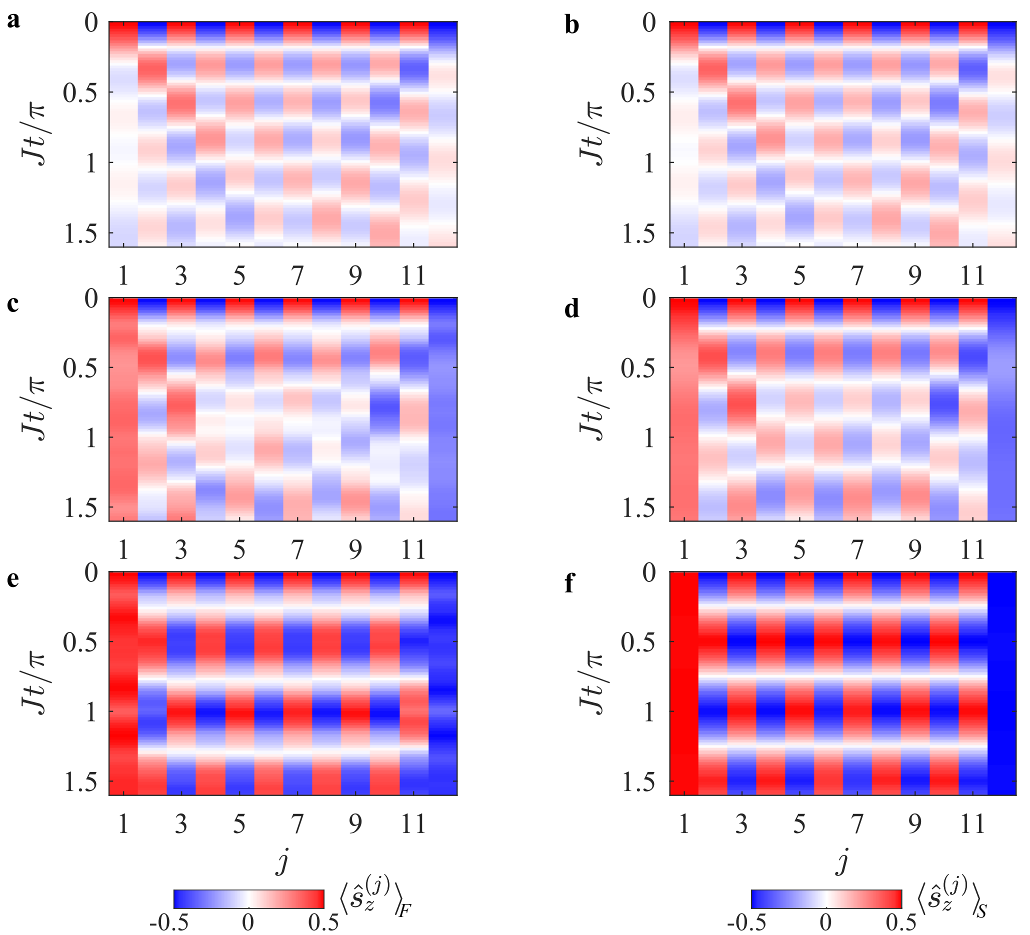

Supplementary Figure 9. **Comparison of SSH spin Hamiltonian and time-dependent Hamiltonian for the staggered-spin configuration**. **(a,c,e)**: Numerical simulation of the short-range spin dynamics using the full time-dependent Hamiltonian (with time dependent Floquet fields), corresponding to subfigures **(j,k,l)** in Supplementary Figure 5. **(b,d,f)**: Numerical simulation using the static SSH Hamiltonian in Eq. 1 for the same parameters. Both models yield similar results, indicating that the contribution of fast-oscillating terms to the dynamics is minimal under our experimental conditions.

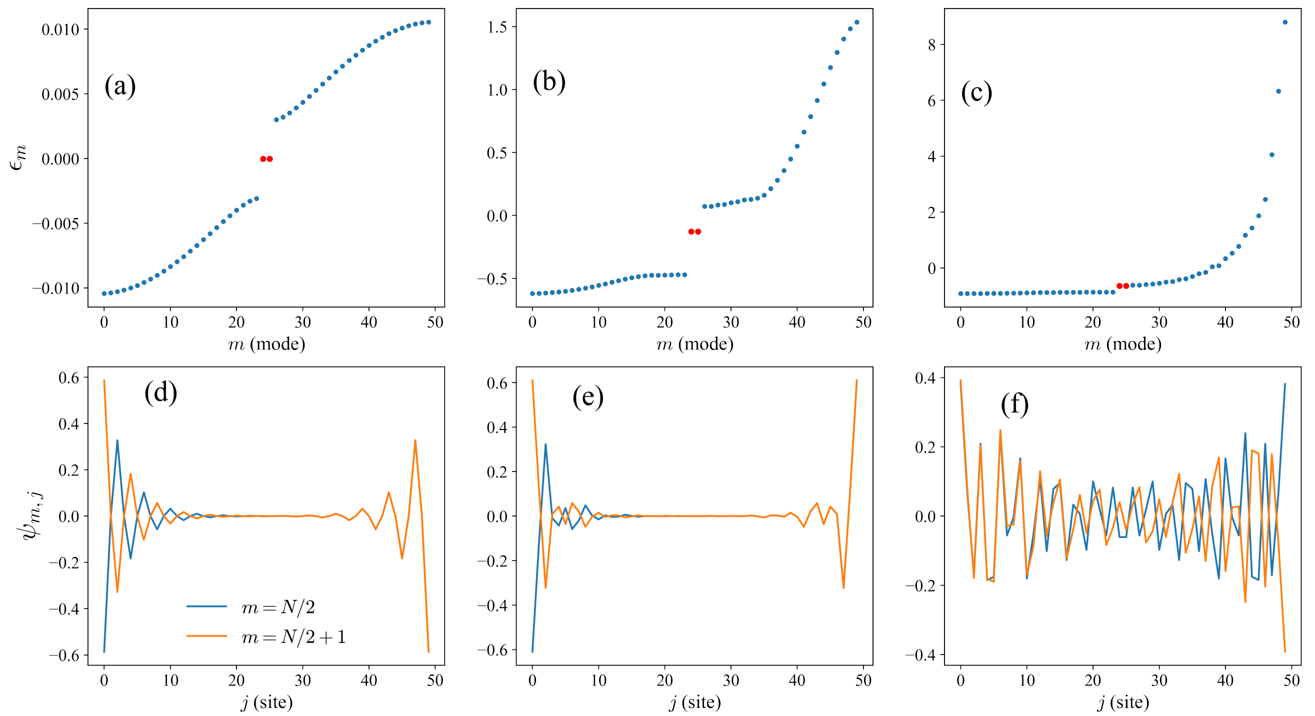

Supplementary Figure 10. **Sub-lattice structure of the long-range SSH model**. We consider the eigensystem of the interaction matrix, $\mathcal{J}\psi_m = \epsilon_m \psi_m$ in the presence of inversion symmetry. **(a - c)** Eigenvalues of $\mathcal{J}$ with interaction range $\xi = 0.1$ **(a)**, $\xi = 2$ **(b)** and $\xi = 6$ (c) [see Eq. (12)], in a crystal of $L = 50$ spins, moderate dimerization $\bar{\eta} = 0.6$ and $\phi = \frac{3\pi}{4}$. **(d - f)** Wavefunction $\psi_{m,j}$ of mid-gap states with index $m = L/2, L/2 + 1$ for the values of ξ listed above. At shorter interaction ranges these states are localized near the edges, while at longer range they are strongly mixed with the bulk.
